# Prediction of Exchange-Correlation Energy of Graphene Sheets from Reverse Degree-Based Molecular Descriptors with Applications

**DOI:** 10.3390/ma15082889

**Published:** 2022-04-14

**Authors:** Mohammed Albadrani, Parvez Ali, Waleed H. El-Garaihy, Hassan Abd El-Hafez

**Affiliations:** 1 Department of Mechanical Engineering, College of Engineering, Qassim University, Unaizah 56452, Saudi Arabia; p.ali@qu.edu.sa (P.A.); w.nasr@qu.edu.sa (W.H.E.-G.); 2 Mechanical Engineering Department, Faculty of Engineering, Suez Canal University, Ismailia 41522, Egypt; 3 Production Engineering and Mechanical Design Department, Faculty of Engineering, Port-Said University, Port-Said 42523, Egypt

**Keywords:** exchange-correlation energy, reverse topological descriptors, graphene

## Abstract

Over the past few years, the popularity of graphene as a potential 2D material has increased since graphene-based materials have applications in a variety of fields, including medicine, engineering, energy, and the environment. A large number of graphene sheets as well as an understanding of graphene’s structural hierarchy are critical to the development of graphene-based materials. For a variety of purposes, it is essential to understand the fundamental structural properties of graphene. Molecular descriptors were used in this study to investigate graphene sheets’ structural behaviour. Based on our findings, reverse degree-based molecular descriptors can significantly affect the exchange-correlation energy prediction. For the exchange-correlation energy of graphene sheets, a linear regression analysis was conducted using the reverse general inverse sum indeg descriptor, $RG_{ISI_{\left(p,q\right)}}$. From $RG_{ISI_{\left(p,q\right)}}$, a set of reverse topological descriptors can be obtained all at once as a special case, resulting in a model with a high correlation coefficient (*R* between 0.896 and 0.998). Used together, these reverse descriptors are graphed in relation to their response to graphene. Based on this study’s findings, it is possible to predict the exchange correlation energy as well as the geometric structures of graphene sheets with very little computational cost.

## 1. Introduction

Carbon is a widely studied and influential element across many scientific disciplines. Many allotropes of carbon exist, each with special properties, such as graphite, diamond, and amorphous carbon as well as fullerenes, carbon nanotubes (CNTs), and graphene [1,2,3,4,5,6]. Graphene is at the forefront of research in fields such as physics, chemistry, and materials science, among many others. Researchers have been intrigued by graphene due to its great mechanical, transportable, optical, and thermal properties as well as its thermal stability and unique electronic structures [7,8,9]. Graphene is packed in a unique two-dimensional nano-carbon hexagonal lattice [10,11]. Graphene’s unique combination of characteristics strongly qualifies it for use in multiple applications, such as biosensors [9], membranes [12], drug delivery, tissue engineering, sensing applications [13], photodetectors [14], electrochemical sensors [15], and hydrogen-based energy storage [16].

A nanostructure is composed of distinct and measurable elements, known as nano-patterns. In contrast to random patterns, these patterns follow the order of chemical and physical laws. Physical and chemical laws determine how atoms and molecules form discrete and measurable geometric structures, ranging from repeating lattices to complex shapes. Rules from the chemical graph theory can be used to analyze and predict the properties of these well-defined structures [17]. In the chemical graph theory, a chemical structure is represented by a corresponding molecular graph, where vertices represent atoms and edges represent bonds [18]. Molecular descriptors are commonly used in the chemical graph theory to predict various properties of chemical structures. Among the many molecular descriptors available, the topological molecular descriptors are a prominent [19,20]. Topological molecular descriptors are used to transform molecular graphs into mathematical models as well as encrypt significant amounts of information about the molecular structure. Topological molecular descriptors can be classified into a number of groups according to their graph parameters. Some of the well-known topological descriptors include distance [21], degree [22], eccentricity [23], and spectrum-based descriptors [24]. Researchers often prefer degree-based topological descriptors due to their simplicity, and some of the most popular degree-based topological descriptors are the first and second Zagreb [25], Randić [26], sum−connectivity [27], and geometric−arithmetic descriptors [28], etc. Wei et al. [29] recently introduced many reverse degree-based topological descriptors, inspired by their work on degree-based topological descriptors.

In this article, molecular graphs are represented by Ǥ. $\Phi_{u}$ denotes the degree of a vertex u, and Δ(Ǥ) is the maximum degree of the graph Ǥ. The reverse degree [30] of a vertex u is defined as $R_{u}=\Delta \left(Ǥ\right)-\Phi_{u}+1$.

To derive a set of reverse degree-based topological descriptors, we first define the reverse general inverse sum indeg descriptor, denoted by $RG_{ISI_{\left(p,q\right)}}\left(Ǥ\right)$, as follows: $RG_{ISI_{\left(p,q\right)}}\left(Ǥ\right)=\underset{uv\in E\left(G\right)}{\sum }{\left[\Phi_{u}\Phi_{v}\right]}^{p\;}{\left[\Phi_{u}+\Phi_{v}\right]}^{q}\;$ where p and q are any real numbers.

Table 1 Some reverse degree-based topological descriptors derived from the reverse general inverse sum indeg descriptor by assigning specific values to the parameters p and q.

Our main objective is to offer an alternate method, with high accuracy, for computing the exchange-correlation energies of graphene sheets. The DFT calculations of the exchange-correlation energies of graphene sheets have the advantage of being accurate, but they also have the disadvantage of being computationally expensive. Therefore, Section 2 provides a relationship between the exchange-correlation energy of graphene sheets and reverse degree-based topological descriptors. Section 3 contains detailed analytical results for graphene using reverse degree topological descriptors and polynomial as well as numerical comparisons.

## 2. Relationship between the Exchange-Correlation Energy and the Reverse General Inverse Sum Indeg Descriptor of Graphene Sheets

A wide range of molecular descriptors have been proposed in the current literature, but many of them show little evidence that they correlate with any of the physical or chemical properties of the chemical structure. This section highlights the inquiry that was undertaken to determine whether reverse general inverse sum indeg descriptors possess any predictive power and whether or not they should be used in any chemical applications. In order to achieve this, we selected ten graphene sheets from one cycle to ten cycles. The molecular structures of these graphene sheets are provided in Table 2. The exchange-correlation energies (ECE) of these graphene sheets were obtained from the literature [31] and have been listed in Table 2.

The reverse general inverse sum indeg descriptors of these graphene sheets were obtained through direct calculations using the edge partition technique. For example, the reverse general inverse sum indeg descriptor for the graphene sheet *C*_24_ ($RG_{ISI_{\left(p,q\right)}}\left(C_{24}\right)$), shown in Table 2, was obtained by the following way: The molecular graph of *C*_24_ had 24 vertices, 30 edges, and $\Delta \left(C_{24}\right)=3$. Based on the reverse degrees of each of the vertices, the edges set of $C_{24}$ was partitioned into three sets: $rm_{11}\left(C_{24}\right),rm_{12}\left(C_{24}\right),rm_{22}\left(C_{24}\right)$ with cardinalities $\left|rm_{11}\left(C_{24}\right)\right|=12,$ $\left|rm_{12}\left(C_{24}\right)\right|=12,$ $\left|rm_{22}\left(C_{24}\right)\right|=6$. From the definition of a reverse general inverse sum indeg descriptor, we used the following:

$$\begin{array}{l} RG_{ISI_{\left(p,q\right)}}\left(C_{24}\right)=\sum_{uv\in E\left(C_{24}\right)}{\left[\Phi_{u}\Phi_{v}\right]}^{p\;}{\left[\Phi_{u}+\Phi_{v}\right]}^{q}\\ \;\;\;\;\;\;\;=12{\left[\left(1\right)\left(1\right)\right]}^{p}{\left[1+1\right]}^{q}+12{\left[\left(1\right)\left(2\right)\right]}^{p}{\left[1+2\right]}^{q}+{\left[\left(2\right)\left(2\right)\right]}^{p}{\left[2+2\right]}^{q} \end{array}$$

After simplification, we arrived at $RG_{ISI_{\left(p,q\right)}}\left(C_{24}\right)=3{\left[2\right]}^{q+2}+{\left[2\right]}^{p+2}{\left[3\right]}^{q+1}+6{\left[4\right]}^{p+q}$.

Table 3 lists 11 reverse topological descriptors: the reverse first and second Zagreb descriptor, the reverse Randić descriptor, the reverse sum−connectivity descriptor, the reverse harmonic descriptor, the reverse hyper Zagreb descriptor, the reverse geometric−arithmetic descriptor, the reverse arithmetic−geometric descriptor, the reverse inverse sum indeg descriptor, the reverse redefined first Zagreb descriptor, and the reverse redefined third Zagreb descriptor. These descriptors were obtained by setting specific values of *p* and *q*, such as the following:

$\left(0,1\right),\left(1,0\right),\left(\frac{-1}{\;2},0\right),\left(0,\;\frac{-1}{\;2}\right),\left(0,-1\right),\left(0,2\right),\left(\frac{1}{2},-1\right),$ $\left(\frac{-1}{\;2},1\right),\left(1,-1\right),\left(-1,1\right),\left(1,1\right)$ in the reverse general inverse sum indeg descriptors (Table 1) for each graphene sheet from C_6_ to *C*_24_.

**Table 3:** Values of the reverse topological descriptors from *C*6 to *C*32.

(p,q)	(0,1)	(1,0)	$\left(\frac{-1}{\;\;\;2},0\right)$	$\left(0,\;\frac{-1}{\;\;\;2}\right)$	(0,−1)	(0,2)	$\left(\frac{1}{2},-1\right)$	$\left(\frac{-1}{\;\;\;2},1\right)$	(1,−1)	(−1,1)	(1,1)
*Graphene Sheets*	$RM_{1}$	$RM_{2}$	$RR_{\frac{1}{2}}$	RSCI	RH	RHZ	RGA	RAG	RISI	$RReZG_{1}$	$RReZG_{3}$
*C*_6_	12	6	6	4.2426	6	24	6	6	3	12	12
*C*_10_	38	33	6.8284	6.0165	6.6667	136	10.771	11.243	9.1667	14	122
*C*_13_	48	39	10.243	8.5855	10	162	14.657	15.364	11.5	21	138
*C*_16_	58	45	13.657	11.154	13.333	188	18.542	19.485	13.833	28	154
*C*_19_	68	51	17.071	13.723	16.667	214	22.428	23.606	16.167	35	170
*C*_22_	79	60	20.364	16.267	20	249	26.485	27.546	19	41.5	202
*C*_24_	84	60	23.485	18.414	23	252	29.314	30.728	20	48	192
*C*_28_	98	71	27.485	21.534	27	296	34.314	35.728	23.5	56	230
*C*_30_	104	73	30.399	24.104	30.33	306	37.199	38.849	24.833	62	230
*C*_32_	110	75	33.314	25.673	32.667	316	40.97	41.97	26.167	68	230

**Figure 1:**
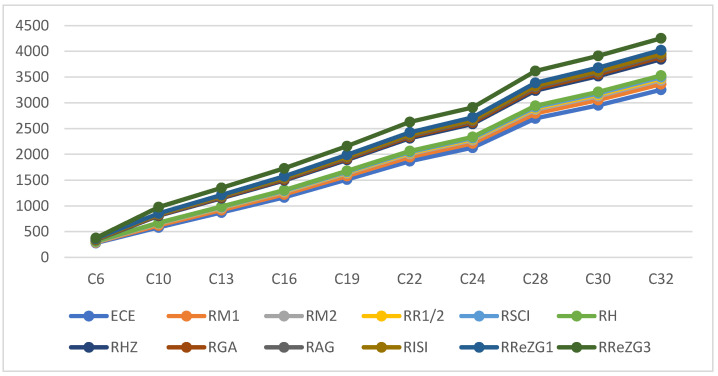
Variation of reverse topological descriptors and ECE.

**Figure 2:**
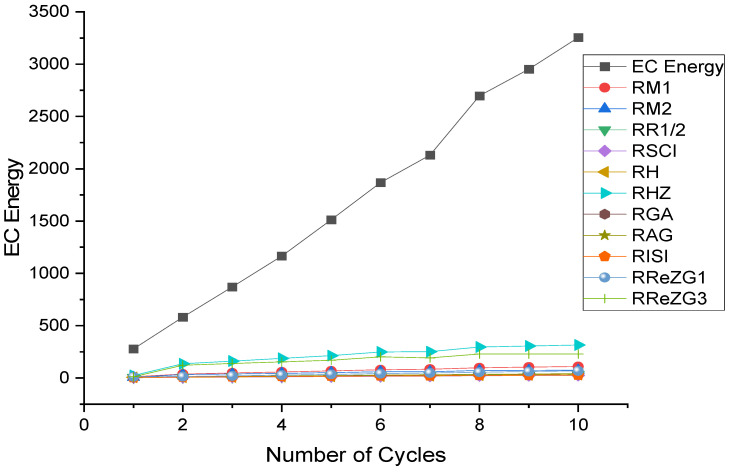
ECE and reverse descriptors.

**Figure 3:**
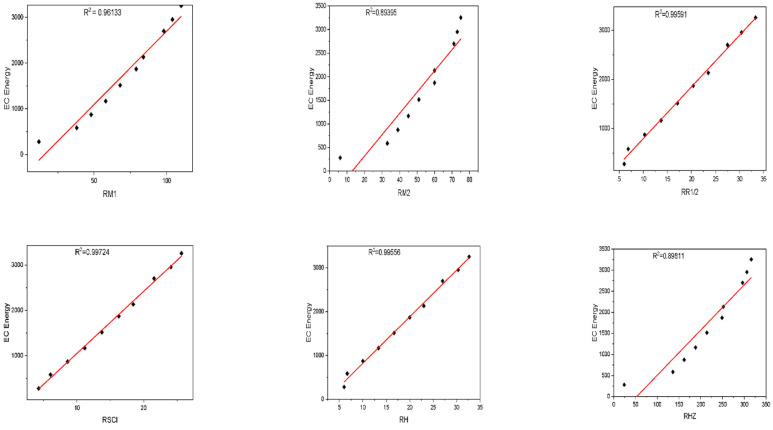

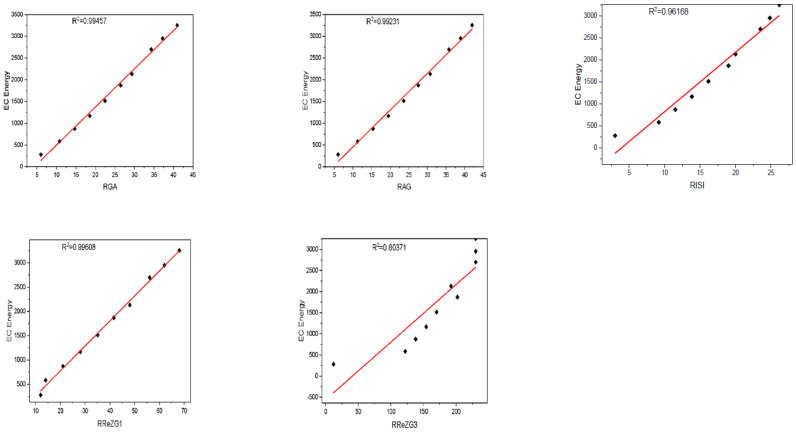
Plot between the reverse topological descriptors and ECE of graphene sheets from *C*_6_ to *C*_32_.

**Figure 4:**
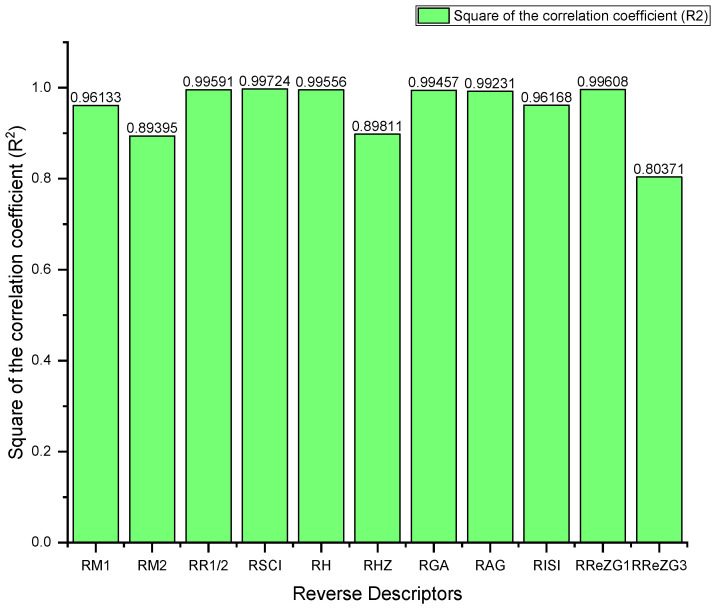
Predictive potential of the reverse topological descriptors via square of the correlation coefficient $R^{2}$.

To predict the exchange-correlation energy of the graphene sheets, the following linear regression model was used:

$$ECE=\alpha \left(RG_{ISI_{\left(p,q\right)}}\right)+\beta ,$$

where ECE is the exchange-correlation energy of the graphene sheets from *C*_6_ to *C*_32_, β is the regression model constant, α is the reverse topological descriptor coefficient, and $RG_{ISI_{\left(p,q\right)}}$ is any predictor from Table 1. This linear regression model was used in compiling Table 4, which used SPSS software to show the regression equations of the 11 reverse topological descriptors, the correlation coefficient between the exchange-correlation energy of the graphene sheets, and the reverse topological descriptors from the data obtained from Table 2 and Table 3. Statistical quantities, such as the standard error (SE) and the F-test, were used to check the reliability of the predictive models listed in Table 4. Based on Table 2 and Table 3, we found that the reverse topological descriptors and the exchange-correlation energy exhibit similar trends and Figure 1 and Figure 2 illustrates this similarity. Figure 3 shows the linear relationship between the exchange-correlation energy while Figure 4 graphically depicts the predictive potential of the reverse topological descriptors via the square of the correlation $\left(R^{2}\right)$ with the help of the reverse topological descriptors of the studied graphene sheets using the regression model presented in Table 4.

## 3. Reverse General Inverse Sum Indeg Descriptor of Graphene

**Figure 5:**
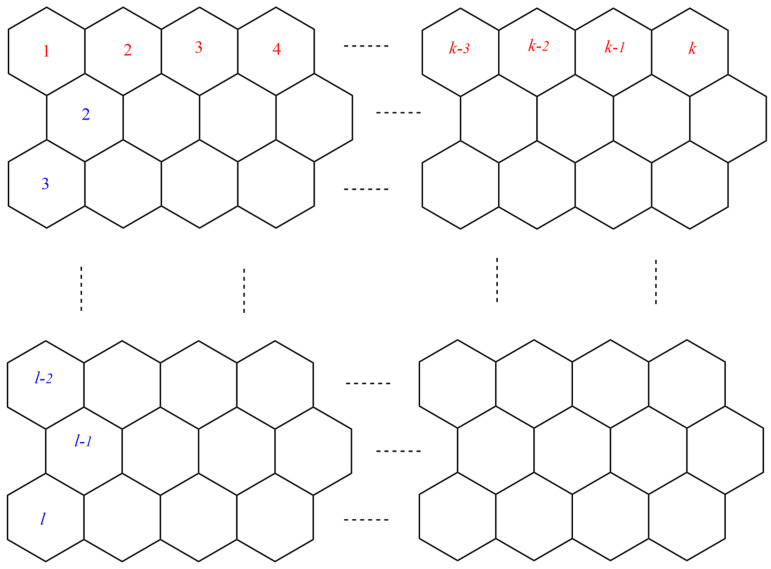
Graphene structure with l>1,k>1.

**Figure 6:**
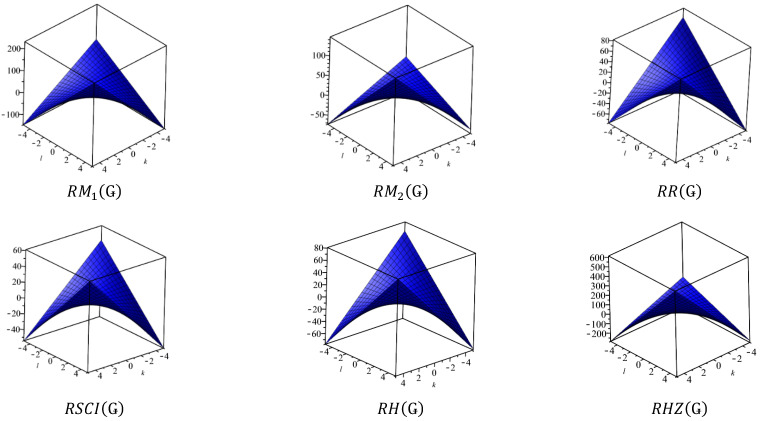

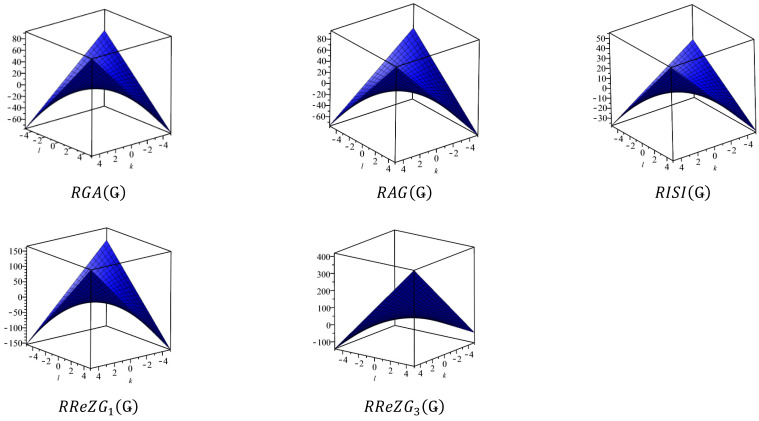
3D Plots when ( l>1,k>1).

**Figure 7:**
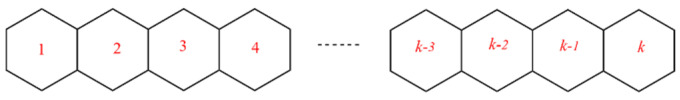
Graphene structure with l=1,k>1.

**Figure 8:**
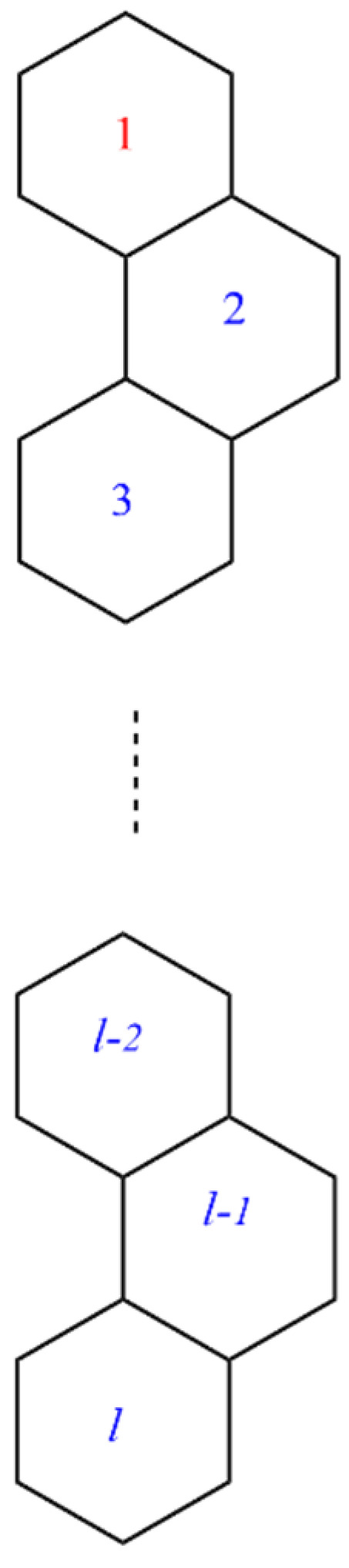
Graphene structure with l>1,k=1.

**Figure 9:**
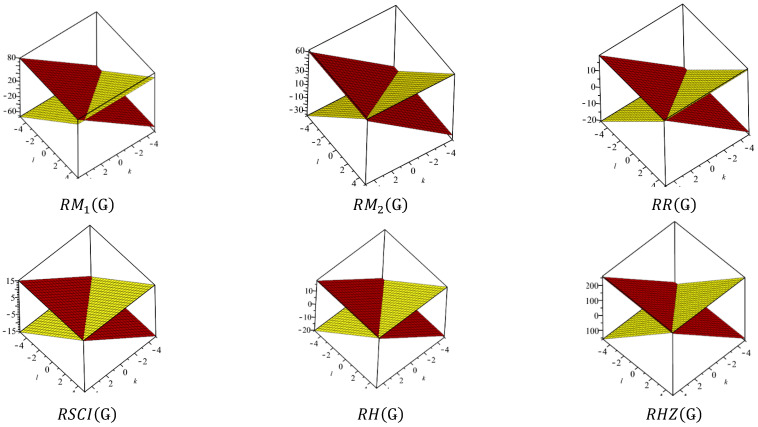

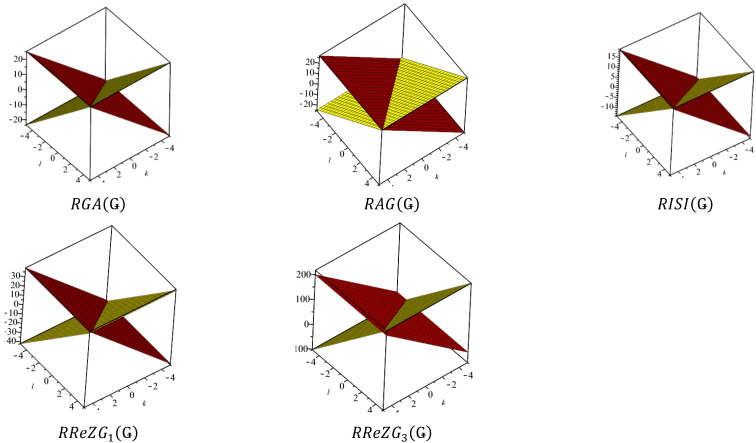
3D plots when ( l=1,k>1) and ( l>1, k=1).

**Figure 10:**
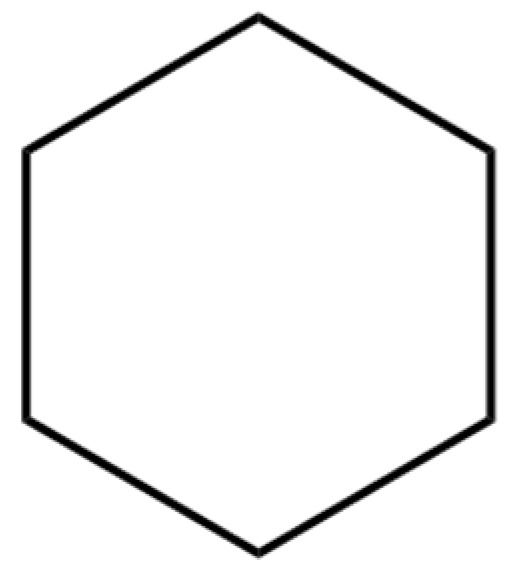
Graphene structure with l=1,k=1.

This section covers graphene systems, which have gained a lot of research interest across a wide range of applications due to their fascinating properties. There are numerous studies [32,33,34,35,36,37,38,39,40] dedicated to the computation of topological descriptors of graphene systems in recent years. Most of these studies are devoted to obtaining an individual formula for each topological descriptor. This article presents a general reverse degree-based topological descriptor, namely, a reverse general inverse sum indeg descriptor from which 11 other reverse degree-based topological descriptors can be obtained. To compute the general reverse inverse sum indeg descriptor for the molecular structure of the graphene under study, we considered four different cases based on the number of rows (l) and the number of benzene rings in each row (k). Initially, the case in which the number of rows and the number of rings in each row were both greater than one was considered, as shown in Figure 5 and Figure 6 as 3D plots. For the second case, the graphene structure had only one row and more than one benzene ring. Figure 7 shows such a situation. In the third case, there was more than one row with only one benzene ring in each column, as shown in Figure 8 and Figure 9 as 3D plots. Figure 10 represents the last case where there was only one benzene ring. Using these four cases and edge partitioning as well as degree counting and graph structure analysis, the reverse general inverse sum indeg descriptor of graphene (Ǥ) was derived as follows:

**Theorem** **1.** *The reverse general inverse sum indeg descriptor $RG_{ISI_{\left(p,q\right)}}\left(Ǥ\right)$of graphene is as follows:*

$$RG_{ISI_{\left(p,q\right)}}\left(Ǥ\right)=\{\left(3lk-2k-l-1\right){\left[1\right]}^{p}{\left[2\right]}^{q}+\left(4k+2l-4\right){\left[2\right]}^{p}{\left[3\right]}^{q}+\left(l+4\right){\left[4\right]}^{p+q},$$

           if l>1,k>1

$$and\;RG_{ISI_{\left(p,q\right)}}\left(Ǥ\right)=\left(k-1\right){\left[1\right]}^{p}{\left[2\right]}^{q}+\left(4k-4\right){\left[2\right]}^{p}{\left[3\right]}^{q}+\left(6\right){\left[4\right]}^{p+q},if\;l=1,k>1$$

$$and\;RG_{ISI_{\left(p,q\right)}}\left(G\right)=\left(2l-3\right){\left[1\right]}^{p}{\left[2\right]}^{q}+\left(2l\right){\left[2\right]}^{p}{\left[3\right]}^{q}+\left(l+4\right){\left[4\right]}^{p+q},\;if\;l>1,\;k=1$$

$$and\;RG_{ISI_{\left(p,q\right)}}\left(G\right)=\left(6\right){\left[2\right]}^{q},\;\mathrm{if}\;l=1,\;k=1$$

**Proof.**  The proof was built by taking the four cases into account. □

**Case** **1.**  *From the graph structure analysis, the reverse edge partition of graphene when*l>1,k>1*contained*$rm_{1,1}=3lk-2k-l-1$*edges*, $rm_{1,2}=4k+2l-4$*edges*, *and*$rm_{2,2}=l+4$*edges*.

Then, applying the definition of the reverse general inverse sum indeg descriptor, $RG_{ISI_{\left(p,q\right)}}\left(Ǥ\right)$, we arrived at $RG_{ISI_{\left(p,q\right)}}\left(Ǥ\right)=\underset{uv\in E\left(Ǥ\right)}{\sum }{\left[\Phi_{u}\Phi_{v}\right]}^{p\;}{\left[\Phi_{u}+\Phi_{v}\right]}^{q}$

$$RG_{ISI_{\left(p,q\right)}}\left(Ǥ\right)=\underset{uv\in E_{1}\left(Ǥ\right)}{\sum }{\left[\Phi_{u}\Phi_{v}\right]}^{p\;}{\left[\Phi_{u}+\Phi_{v}\right]}^{q}+\underset{uv\in E_{2}\left(Ǥ\right)}{\sum }{\left[\Phi_{u}\Phi_{v}\right]}^{p\;}{\left[\Phi_{u}+\Phi_{v}\right]}^{q}$$

$$+\underset{uv\in E_{2}\left(Ǥ\right)}{\sum }{\left[\Phi_{u}\Phi_{v}\right]}^{p\;}{\left[\Phi_{u}+\Phi_{v}\right]}^{q}$$

$$=rm_{1,1}{\left[\left(1\right)\left(1\right)\right]}^{p}{\left[1+1\right]}^{q}+rm_{1,2}{\left[\left(1\right)\left(2\right)\right]}^{p}{\left[1+2\right]}^{q}+rm_{2,2}{\left[\left(2\right)\left(2\right)\right]}^{p}{\left[2+2\right]}^{q}$$

(1) $$=\left(3lk-2k-l-1\right){\left[1\right]}^{p}{\left[2\right]}^{q}+\left(4k+2l-4\right){\left[2\right]}^{p}{\left[3\right]}^{q}+\left(l+4\right){\left[4\right]}^{p+q}$$

Using Table 1, in Equation (1), the following 11 reverse topological descriptors for the graphene when l>1,k>1 were obtained.

**Remark** **1.**

(i)
$RG_{ISI_{\left(0,1\right)}}=RM_{1}\left(Ǥ\right)=\left(3lk-2k-l-1\right){\left[2\right]}^{1}+\left(4k+2l-4\right){\left[3\right]}^{1}+\left(l+4\right){\left[4\right]}^{1}$
$$RM_{1}\left(Ǥ\right)=6lk+8k+8l+2$$
(ii)
$RG_{ISI_{\left(1,0\right)}}=RM_{2}\left(Ǥ\right)=\left(3lk-2k-l-1\right){\left[1\right]}^{1}+\left(4k+2l-4\right){\left[2\right]}^{1}+\left(l+4\;\right){\left[4\right]}^{1}$
$$RM_{2}\left(Ǥ\right)=3lk+6k+7l+7$$
(iii)
$RG_{ISI_{\left(\frac{-1}{\;2},0\right)}}=RR\left(Ǥ\right)=\left(3lk-2k-l-1\right)+\left(4k+2l-4\right)\frac{1}{\sqrt{2}}+\left(l+4\right)\frac{1}{2}\;$

(iv)
$RG_{ISI_{\left(0,\frac{-1}{\;2}\right)}}=RSCI\left(Ǥ\right)=\left(3lk-2k-l-1\right)\frac{1}{\sqrt{2}}+\left(4k+2l-4\right)\frac{1}{\sqrt{3}}+\left(l+4\right)\frac{1}{2}$

(v)
$2RG_{ISI_{\left(0,-1\right)}}=RH\left(Ǥ\right)=2\left(3lk-2k-l-1\right){\left[2\right]}^{-1}+2\left(4k+2l-4\right){\left[3\right]}^{-1}+2\left(l+4\right){\left[4\right]}^{-1}$
$$RH\left(Ǥ\right)=\left(3lk-2k-l-1\right)+\left(4k+2l-4\right)\frac{2}{3}+\left(l+4\right)\frac{1}{2}$$
(vi)
$RG_{ISI_{\left(0,2\right)}}=RHZ\left(G\right)=\left(3lk-2k-l-1\right){\left[2\right]}^{2}+\left(4k+2l-4\right){\left[3\right]}^{2}+\left(l+4\right){\left[4\right]}^{2}\;$
RHZ(Ǥ)=12lk+28k+30l+24
(vii)
$\;2RG_{ISI_{\left(\frac{1}{2},-1\right)}}=RGA\left(G\right)=2\left(3lk-2k-l-1\right){\left[1\right]}^{\frac{1}{2}}{\left[2\right]}^{-1}+2\left(4k+2l-4\right){\left[2\right]}^{\frac{1}{2}}{\left[3\right]}^{-1}+2\left(l+4\right){\left[4\right]}^{\frac{1}{2}}{\left[4\right]}^{-1}\;$
$$RGA\left(Ǥ\right)=\left(3lk-2k-l-1\right)+\left(4k+2l-4\right)\frac{2\sqrt{2}}{3}+\left(l+4\right)$$
(viii)
$\frac{1}{2}RG_{ISI_{\left(\frac{-1}{\;2},1\right)}}=RAG\left(Ǥ\right)=\left(3lk-2k-l-1\right)+\left(4k+2l-4\right)\frac{1}{2}{\left[2\right]}^{\frac{-1}{2}}{\left[3\right]}^{1}+\left(l+4\right)\frac{1}{2}{\left[4\right]}^{\frac{-1}{2}}{\left[4\right]}^{1}$
$$RAG\left(Ǥ\right)=\left(3lk-2k-l-1\right)+\left(4k+2l-4\right)\frac{3}{2\sqrt{2}}+\left(l+4\right)$$
(ix)
$\;RG_{ISI_{\left(1,-1\right)}}=RISI\left(Ǥ\right)=\left(3lk-2k-l-1\right){\left[1\right]}^{1}{\left[2\right]}^{-1}+\left(4k+2l-4\right){\left[2\right]}^{1}{\left[3\right]}^{-1}+\left(l+4\right){\left[4\right]}^{1}{\left[4\right]}^{-1}$
$$RISI\left(Ǥ\right)=\left(3lk-2k-l-1\right)\frac{1}{2}+\left(4k+2l-4\right)\frac{2}{3}+\left(l+4\right)$$
(x)
$RG_{ISI_{\left(-1,1\right)}}=RReZG_{1}\left(Ǥ\right)=\left(3lk-2k-l-1\right){\left[1\right]}^{-1}{\left[2\right]}^{1}+\left(4k+2l-4\right){\left[2\right]}^{-1}{\left[3\right]}^{1}+\left(l+4\right){\left[4\right]}^{-1}{\left[4\right]}^{1}$
$$RReZG_{1}\left(Ǥ\right)=\left(3lk-2k-l-1\right)2+\left(4k+2l-4\right)\frac{3}{2}+\left(l+4\right)$$
(xi)
$RG_{ISI_{\left(1,1\right)}}=RReZG_{3}\left(Ǥ\right)=\left(3lk-2k-l-1\right){\left[1\right]}^{1}{\left[2\right]}^{1}+\left(4k+2l-4\right){\left[2\right]}^{1}{\left[3\right]}^{1}+\left(l+4\;\right){\left[4\right]}^{1}{\left[4\right]}^{1}$
$$RReZG_{3}\left(Ǥ\right)=6lk+20k+26l+38$$

**Case** **2.**  When l=1,k>1, the reverse edge partition of graphene contained $rm_{1,1}=k-1$ edges, $rm_{1,2}=4k-4$ edges, and $rm_{2,2}=6$ edges.

Applying the definition of the reverse general inverse sum indeg descriptor, $RG_{ISI_{\left(p,q\right)}}\left(Ǥ\right)$,

$$RG_{ISI_{\left(p,q\right)}}\left(Ǥ\right)=\underset{uv\in E\left(Ǥ\right)}{\sum }{\left[\Phi_{u}\Phi_{v}\right]}^{p\;}{\left[\Phi_{u}+\Phi_{v}\right]}^{q}$$

$$RG_{ISI_{\left(p,q\right)}}\left(Ǥ\right)=\underset{uv\in E_{1}\left(Ǥ\right)}{\sum }{\left[\Phi_{u}\Phi_{v}\right]}^{p\;}{\left[\Phi_{u}+\Phi_{v}\right]}^{q}+\underset{uv\in E_{2}\left(Ǥ\right)}{\sum }{\left[\Phi_{u}\Phi_{v}\right]}^{p\;}{\left[\Phi_{u}+\Phi_{v}\right]}^{q}$$

$$+\underset{uv\in E_{3}\left(Ǥ\right)}{\sum }{\left[\Phi_{u}\Phi_{v}\right]}^{p\;}{\left[\Phi_{u}+\Phi_{v}\right]}^{q}$$

$$=rm_{1,1}{\left[\left(1\right)\left(1\right)\right]}^{p}{\left[1+1\right]}^{q}+rm_{1,2}{\left[\left(1\right)\left(2\right)\right]}^{p}{\left[1+2\right]}^{q}+rm_{2,2}{\left[\left(2\right)\left(2\right)\right]}^{p}{\left[2+2\right]}^{q}$$

(2) $$=\left(k-1\right){\left[1\right]}^{p}{\left[2\right]}^{q}+\left(4k-4\right){\left[2\right]}^{p}{\left[3\right]}^{q}+\left(6\right){\left[4\right]}^{p+q}$$

Using Table 1, in Equation (2), we noted the following 11 reverse topological descriptors for the graphene when l=1,k>1

**Remark** **2.**

(i)
$RG_{ISI_{\left(0,1\right)}}=RM_{1}\left(Ǥ\right)=\left(k-1\right){\left[1\right]}^{0}{\left[2\right]}^{1}+\left(4k-4\right){\left[2\right]}^{0}{\left[3\right]}^{1}+\left(6\right){\left[4\right]}^{0}{\left[4\right]}^{1}\;$
$$RM_{1}\left(G\right)=14k+10$$
(ii)
$RG_{ISI_{\left(1,0\right)}}=RM_{2}\left(Ǥ\right)=\left(k-1\right){\left[1\right]}^{1}{\left[2\right]}^{0}+\left(4k-4\right){\left[2\right]}^{1}{\left[3\right]}^{0}+\left(6\right){\left[4\right]}^{1}{\left[4\right]}^{0}$
$$RM_{2}\left(Ǥ\right)=9k+15$$
(iii)
$RG_{ISI_{\left(\frac{-1}{\;2},0\right)}}=RR\left(Ǥ\right)=\left(k-1\right){\left[1\right]}^{\frac{-1}{\;2}}{\left[2\right]}^{0}+\left(4k-4\right){\left[2\right]}^{\frac{-1}{\;2}}{\left[3\right]}^{0}+\left(6\right){\left[4\right]}^{\frac{-1}{\;2}}{\left[4\right]}^{0}$
$$RR\left(Ǥ\right)=\left(1+2\sqrt{2}\right)k+\left(3-\frac{4}{\sqrt{2}}\right)$$
(iv)
$RG_{ISI_{\left(0,\frac{-1}{\;2}\right)}}=RSCI\left(Ǥ\right)=\left(k-1\right){\left[1\right]}^{0}{\left[2\right]}^{\frac{-1}{\;2}}+\left(4k-4\right){\left[2\right]}^{0}{\left[3\right]}^{\frac{-1}{\;2}}+\left(6\right){\left[4\right]}^{0}{\left[4\right]}^{\frac{-1}{\;2}}$
$$RSCI\left(Ǥ\right)=\left(\frac{1}{\sqrt{2}}+\frac{4}{\sqrt{3}}\right)k+\left(3-\frac{1}{\sqrt{2}}-\frac{4}{\sqrt{3}}\right)$$
(v)
$2RG_{ISI_{\left(0,-1\right)}}=RH\left(Ǥ\right)=\left(k-1\right){\left[1\right]}^{0}{\left[2\right]}^{-1}+\left(4k-4\right){\left[2\right]}^{0}{\left[3\right]}^{-1}+\left(6\right){\left[4\right]}^{0}{\left[4\right]}^{-1}$
$$RH\left(Ǥ\right)=\frac{11}{3}k-\frac{2}{3}$$
(vi)
$RG_{ISI_{\left(0,2\right)}}=RHZ\left(Ǥ\right)=\left(k-1\right){\left[1\right]}^{0}{\left[2\right]}^{2}+\left(4k-4\right){\left[2\right]}^{0}{\left[3\right]}^{2}+\left(6\right){\left[4\right]}^{0}{\left[4\right]}^{2}$
RHZ(Ǥ)=40k+56
(vii)
$\;2RG_{ISI_{\left(\frac{1}{2},-1\right)}}=RGA\left(Ǥ\right)=2\left(k-1\right){\left[1\right]}^{\frac{1}{2}}{\left[2\right]}^{-1}+2\left(4k-4\right){\left[2\right]}^{\frac{1}{2}}{\left[3\right]}^{-1}+2\left(6\right){\left[4\right]}^{\frac{1}{2}}{\left[4\right]}^{-1}$
$$RGA\left(Ǥ\right)=\left(\frac{3+8\sqrt{2}}{3}\right)k+\left(5-\frac{8\sqrt{2}}{3}\right).\;$$
(viii)
$\frac{1}{2}RG_{ISI_{\left(\frac{-1}{\;2},1\right)}}=RAG\left(Ǥ\right)=\;\frac{1}{2}\left(k-1\right){\left[2\right]}^{1}+\;\frac{1}{2}\left(4k-4\right){\left[2\right]}^{\frac{-1}{\;2}}{\left[3\right]}^{1}+\;\frac{1}{2}\left(6\;\right){\left[4\right]}^{\frac{-1}{\;2}}{\left[4\right]}^{1}$
$$RAG\left(Ǥ\right)=\left(1+3\sqrt{2}\right)k+\left(5-3\sqrt{2}\right)$$
(ix)
$\;RG_{ISI_{\left(1,-1\right)}}=RISI\left(Ǥ\right)=\left(k-1\right){\left[1\right]}^{1}{\left[2\right]}^{-1}+\left(4k-4\right){\left[2\right]}^{1}{\left[3\right]}^{-1}+\left(6\right){\left[4\right]}^{1}{\left[4\right]}^{-1}$
$$RISI\left(Ǥ\right)=\frac{19}{6}k+\frac{17}{6}$$
(x)
$RG_{ISI_{\left(-1,1\right)}}=RReZG_{1}\left(Ǥ\right)=\left(k-1\right){\left[1\right]}^{-1}{\left[2\right]}^{1}+\left(4k-4\right){\left[2\right]}^{-1}{\left[3\right]}^{1}+\left(6\right){\left[4\right]}^{-1}{\left[4\right]}^{1}$
$$RReZG_{1}\left(Ǥ\right)=8k-\frac{1}{2}$$
(xi)
$RG_{ISI_{\left(1,1\right)}}=RReZG_{3}\left(Ǥ\right)=\left(k-1\right){\left[1\right]}^{1}{\left[2\right]}^{1}+\left(4k-4\right){\left[2\right]}^{1}{\left[3\right]}^{1}+\left(6\right){\left[4\right]}^{1}{\left[4\right]}^{1}$
$$RReZG_{3}\left(Ǥ\right)=26k+70$$

**Case** **3.**  *For*l>1, k=1,*the reverse edge partition of the graphene contains*$rm_{1,1}=2l-3$*edges*, $rm_{1,2}=2l$*edges*, *and*$rm_{2,2}=l+4$*edges*.

Using the definition of the reverse general inverse sum indeg descriptor, $RG_{ISI_{\left(p,q\right)}}\left(Ǥ\right)$,

$$G_{ISI_{\left(p,q\right)}}\left(Ǥ\right)=\underset{uv\in E\left(Ǥ\right)}{\sum }{\left[\Phi_{u}\Phi_{v}\right]}^{p\;}{\left[\Phi_{u}+\Phi_{v}\right]}^{q}$$

$$RG_{ISI_{\left(p,q\right)}}\left(Ǥ\right)=\underset{uv\in E_{1}\left(Ǥ\right)}{\sum }{\left[\Phi_{u}\Phi_{v}\right]}^{p\;}{\left[\Phi_{u}+\Phi_{v}\right]}^{q}+\underset{uv\in E_{2}\left(Ǥ\right)}{\sum }{\left[\Phi_{u}\Phi_{v}\right]}^{p\;}{\left[\Phi_{u}+\Phi_{v}\right]}^{q}$$

$$+\underset{uv\in E_{3}\left(Ǥ\right)}{\sum }{\left[\Phi_{u}\Phi_{v}\right]}^{p\;}{\left[\Phi_{u}+\Phi_{v}\right]}^{q}$$

$$=rm_{1,1}{\left[\left(1\right)\left(1\right)\right]}^{p}{\left[1+1\right]}^{q}+rm_{1,2}{\left[\left(1\right)\left(2\right)\right]}^{p}{\left[1+2\right]}^{q}+rm_{2,2}{\left[\left(2\right)\left(2\right)\right]}^{p}{\left[2+2\right]}^{q}$$

(3) $$=\left(2l-3\right){\left[1\right]}^{p}{\left[2\right]}^{q}+\left(2l\right){\left[2\right]}^{p}{\left[3\right]}^{q}+\left(l+4\right){\left[4\right]}^{p+q}$$

Using Table 1, in Equation (3), we noted the following reverse topological descriptors for the graphene when l>1,k=1

**Remark** **3.**

(i)
$RG_{ISI_{\left(0,1\right)}}=RM_{1}\left(Ǥ\right)=\left(2l-3\right){\left[1\right]}^{0}{\left[2\right]}^{1}+\left(2l\right){\left[2\right]}^{0}{\left[3\right]}^{1}+\left(l+4\right){\left[4\right]}^{0}{\left[4\right]}^{1}$
$$RM_{1}\left(Ǥ\right)=14l-2$$
(ii)
$RG_{ISI_{\left(1,0\right)}}=RM_{2}\left(Ǥ\right)=\left(2l-3\right){\left[1\right]}^{1}{\left[2\right]}^{0}+\left(2l\right){\left[2\right]}^{1}{\left[3\right]}^{0}+\left(l+4\right){\left[4\right]}^{1}{\left[4\right]}^{0}$
$$RM_{2}\left(Ǥ\right)=10l+13$$
(iii)
$RG_{ISI_{\left(\frac{-1}{\;2},0\right)}}=RR\left(Ǥ\right)=\left(2l-3\right){\left[1\right]}^{\frac{-1}{\;2}}{\left[2\right]}^{0}+\left(2l\right){\left[2\right]}^{\frac{-1}{\;2}}{\left[3\right]}^{0}+\left(l+4\right){\left[4\right]}^{\frac{-1}{\;2}}{\left[4\right]}^{0}$
$$RR\left(Ǥ\right)=\left(\frac{5+2\sqrt{2}}{2}\right)l-1$$
(iv)
$RG_{ISI_{\left(0,\frac{-1}{\;2}\right)}}=RSCI\left(Ǥ\right)=\left(2l-3\right){\left[1\right]}^{0}{\left[2\right]}^{\frac{-1}{\;2}}+\left(2l\right){\left[2\right]}^{0}{\left[3\right]}^{\frac{-1}{\;2}}+\left(l+4\right){\left[4\right]}^{0}{\left[4\right]}^{\frac{-1}{\;2}}$
$$RSCI\left(Ǥ\right)=\left(\sqrt{2}+\frac{2}{\sqrt{3}}+\frac{1}{2}\right)l+\left(2-\frac{3}{\sqrt{2}}\right)$$
(v)
$2RG_{ISI_{\left(0,-1\right)}}=RH\left(Ǥ\right)=2\left(2l-3\right){\left[1\right]}^{0}{\left[2\right]}^{-1}+2\left(2l\right){\left[2\right]}^{0}{\left[3\right]}^{-1}+2\left(l+4\right){\left[4\right]}^{0}{\left[4\right]}^{-1}$
$$RH\left(Ǥ\right)=\frac{23}{6}l-1$$
(vi)
$RG_{ISI_{\left(0,2\right)}}=RHZ\left(Ǥ\right)=\left(2l-3\right){\left[1\right]}^{0}{\left[2\right]}^{2}+\left(2l\right){\left[2\right]}^{0}{\left[3\right]}^{2}+\left(l+4\right){\left[4\right]}^{0}{\left[4\right]}^{2}$
RHZ(Ǥ)=42l+52
(vii)
$2RG_{ISI_{\left(\frac{1}{2},-1\right)}}=RGA\left(Ǥ\right)=2\left(2l-3\right){\left[1\right]}^{\frac{1}{2}}{\left[2\right]}^{-1}+2\left(2l\right){\left[2\right]}^{\frac{1}{2}}{\left[3\right]}^{-1}+2\left(l+4\right){\left[4\right]}^{\frac{1}{2}}{\left[4\right]}^{-1}$
$$RGA\left(Ǥ\right)=\left(3+\frac{4\sqrt{2}}{3}\right)l+1$$
(viii)
$\frac{1}{2}RG_{ISI_{\left(\frac{-1}{\;2},1\right)}}=RAG\left(Ǥ\right)=\frac{1}{2}\left(2l-3\right){\left[1\right]}^{\frac{-1}{\;2}}{\left[2\right]}^{1}+\frac{1}{2}\left(2l\right){\left[2\right]}^{\frac{-1}{\;2}}{\left[3\right]}^{1}+\frac{1}{2}\left(l+4\right){\left[4\right]}^{\frac{-1}{\;2}}{\left[4\right]}^{1}$
$$RAG\left(Ǥ\right)=\left(3+\frac{3}{\sqrt{2}}\right)l+1$$
(ix)
$RG_{ISI_{\left(1,-1\right)}}=RISI\left(Ǥ\right)=\left(2l-3\right){\left[1\right]}^{1}{\left[2\right]}^{-1}+\left(2l\right){\left[2\right]}^{1}{\left[3\right]}^{-1}+\left(l+4\right){\left[4\right]}^{1}{\left[4\right]}^{-1}$
$$RISI\left(Ǥ\right)=\frac{10}{3}l+\frac{5}{2}$$
(x)
$RG_{ISI_{\left(-1,1\right)}}=RReZG_{1}\left(Ǥ\right)=\left(2l-3\right){\left[1\right]}^{-1}{\left[2\right]}^{1}+\left(2l\right){\left[2\right]}^{-1}{\left[3\right]}^{1}+\left(l+4\right){\left[4\right]}^{-1}{\left[4\right]}^{1}$
$$RReZG_{1}\left(Ǥ\right)=8l-2$$
(xi)
$RG_{ISI_{\left(1,1\right)}}=RReZG_{3}\left(Ǥ\right)=\left(2l-3\right){\left[1\right]}^{1}{\left[2\right]}^{1}+\left(2l\right){\left[2\right]}^{1}{\left[3\right]}^{1}+\left(l+4\right){\left[4\right]}^{1}{\left[4\right]}^{1}$
$$RReZG_{3}\left(Ǥ\right)=32l+58$$

**Case** **4.**  *When*l=1, k=1,*the reverse edge partition of the graphene contained only*$rm_{1,1}=6$*edges*, *and by the definition of reverse general inverse sum indeg descriptor*, $RG_{ISI_{\left(p,q\right)}}\left(Ǥ\right)$,

$$RG_{ISI_{\left(p,q\right)}}\left(Ǥ\right)=\sum_{uv\in E_{1}\left(Ǥ\right)}{\left[\Phi_{u}\Phi_{v}\right]}^{p\;}{\left[\Phi_{u}+\Phi_{v}\right]}^{q}=rm_{1,1}{\left[\left(1\right)\left(1\right)\right]}^{p}{\left[1+1\right]}^{q}=\left(6\right){\left[1\right]}^{p}{\left[2\right]}^{q}$$

In this case, we noted the following 11 reverse topological descriptors for the graphene as follows:

**Remark** **4.**

(i) $RM_{1}\left(Ǥ\right)=\left(6\right){\left[1\right]}^{0}{\left[2\right]}^{1}=12,$ (ii). $RM_{2}\left(Ǥ\right)=\left(6\right){\left[1\right]}^{1}{\left[2\right]}^{0}=6$
(ii) $RR\left(Ǥ\right)=\left(6\right){\left[1\right]}^{\frac{-1}{\;2}}{\left[2\right]}^{0}=6,$ (iv). $RRSCI\left(Ǥ\right)=\left(6\right){\left[1\right]}^{0}{\left[2\right]}^{\frac{-1}{\;2}}=\frac{6}{\sqrt{2}}$
(iii) $RH\left(Ǥ\right)=2\left(6\right){\left[1\right]}^{0}{\left[2\right]}^{-1}=6,$ (vi). $RHZ\left(Ǥ\right)=\left(6\right){\left[1\right]}^{0}{\left[2\right]}^{2}=24$
(iv) $RGA\left(Ǥ\right)=2\left(6\right){\left[1\right]}^{\frac{1}{2}}{\left[2\right]}^{-1}=6,$ (viii). $RAG\left(Ǥ\right)=\frac{1}{2}\left(6\right){\left[1\right]}^{\frac{-1}{\;2}}{\left[2\right]}^{1}=6$
(v) $RISI\left(Ǥ\right)=\left(6\right){\left[1\right]}^{1}{\left[2\right]}^{-1}=3,$ (x). $RReZG_{1}\left(Ǥ\right)=\left(6\right){\left[1\right]}^{-1}{\left[2\right]}^{1}=12$
(vi) $RReZG_{3}\left(Ǥ\right)=\left(6\right){\left[1\right]}^{1}{\left[2\right]}^{1}=12$

**Figure 11:**
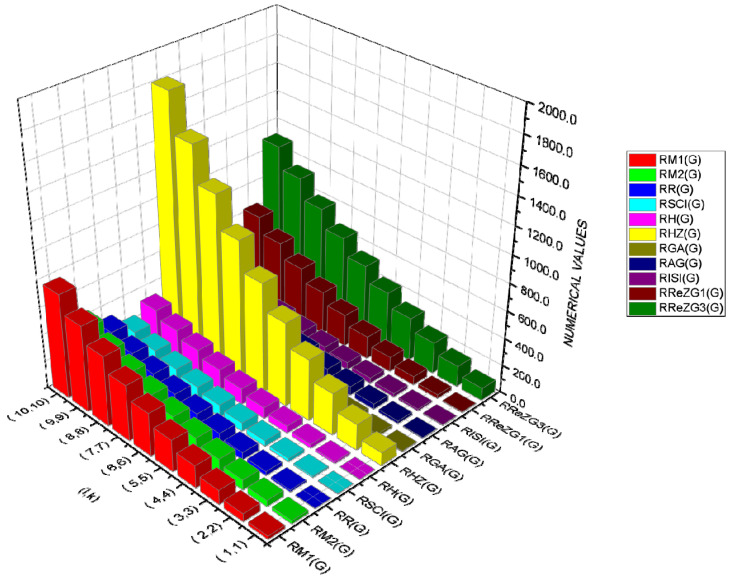
An interactive visualization of Table 5.

In Table 5, the numerical values of the 11 reverse topological descriptors calculated with graphene’s analytical expressions when l>1,k>1 are presented. From Table 5, it is possible to see how individual reverse topological descriptor differ and how they are similar. The computational results show that reverse topological descriptors are highly dependent on the values of *l* and *k*. As these values increase, the magnitude of all reverse descriptors also increases, and this can be visualized by the 3D graphical representation in Figure 11.

## 4. Conclusions

In this paper, we presented a reverse general inverse sum inverse degree descriptor $RG_{ISI_{\left(p,q\right)}}$ from which one can derive a set of reverse degree-based topological descriptors. In order to assess the predictive potential of $RG_{ISI_{\left(p,q\right)}}$, we selected the exchange-correlation energy of the graphene sheets as a data example. Based on the results obtained in this article, we can summarize them as follows:

- The regression models (Table 4) derived from reverse topological descriptors in the present article were extremely accurate for predicting the exchange-correlation energies in the graphene sheets.
- The reverse sum−connectivity descriptor with $R^{2}=0.997$ was the best predictor among the 11 studied descriptors. Meanwhile, the reverse redefined first Zagreb descriptor performed poorly.
- The density functional theory (DFT) calculations of the electronic structure, such as the exchange-correlation energies of the graphene sheets, were precise; however, they were computationally expensive while the reverse topological descriptors models presented herein required minimal computations and provided high levels of accuracy.
- Analytical expressions of the reverse first and second Zagreb descriptor, reverse Randić descriptor, reverse sum−connectivity descriptor, reverse harmonic descriptor, reverse hyper Zagreb descriptor, reverse geometric−arithmetic descriptor, reverse inverse sum indeg descriptor, and reverse redefined first and third Zagreb descriptors have been obtained for graphene structures.
- Researchers who are trying to better understand the behaviour of graphene are likely to find the numerical values and graphical representations presented in this article helpful.

## Figures and Tables

**Table 1:** Some reverse degree-based topological descriptors derived from the reverse general inverse sum indeg descriptor.

(p,q)	$RG_{ISI_{\left(p,q\right)}}$	Corresponding Reverse Topological Descriptors
(0,1)	$RG_{ISI_{\left(0,1\right)}}=RM_{1}\left(Ǥ\right)$	Reverse first Zagreb descriptor
(1,0)	$RG_{ISI_{\left(1,0\right)}}=RM_{2}\left(Ǥ\right)$	Reverse second Zagreb descriptor
$\left(\frac{-1}{2},0\right)$	$RG_{ISI_{\left(\frac{-1}{2},0\right)}}=RR_{1/2}\left(Ǥ\right)$	Reverse Randić descriptor
$\left(0,\;\frac{-1}{2}\right)$	$RG_{ISI_{\left(0,\frac{-1}{2}\right)}}=RSCI\left(Ǥ\right)$	Reverse sum−connectivity descriptor
(0,−1)	$2RG_{ISI_{\left(0,-1\right)}}=RH\left(Ǥ\right)$	Reverse harmonic descriptor
(0,2)	$RG_{ISI_{\left(0,2\right)}}=RHZ\left(Ǥ\right)$	Reverse hyper Zagreb descriptor
$\left(\frac{1}{2},-1\right)$	$2RG_{ISI_{\left(\frac{1}{2},-1\right)}}=RGA\left(Ǥ\right)$	Reverse geometric−arithmetic descriptor
$\left(\frac{-1}{2},1\right)$	$\frac{1}{2}RG_{ISI_{\left(\frac{-1}{2},1\right)}}=RAG\left(Ǥ\right)$	Reverse arithmetic−geometric descriptor
(1,−1)	$RG_{ISI_{\left(1,-1\right)}}=RISI\left(Ǥ\right)$	Reverse inverse sum indeg descriptor
(−1,1)	$RG_{ISI_{\left(-1,1\right)}}=RReZG_{1}\left(Ǥ\right)$	Reverse redefined first Zagreb descriptor
(1,1)	$RG_{ISI_{\left(1,1\right)}}=RReZG_{3}\left(Ǥ\right)$	Reverse redefined third Zagreb descriptor

**Table 2:** Graphene sheets from *C*_6_ to *C*_32_ with their exchange-correlation energy and reverse general inverse sum indeg descriptors.

Graphene Sheets	EC Energy	$\begin{array}{l} RG_{ISI_{\left(p,q\right)}}\left(Ǥ\right)\\ =\sum_{uv\in E\left(G\right)}{\left[\Phi_{u}\Phi_{v}\right]}^{p\;}{\left[\Phi_{u}+\Phi_{v}\right]}^{q} \end{array}$
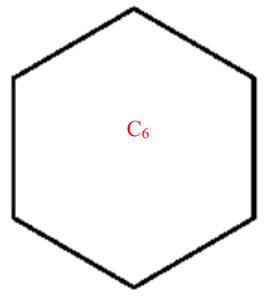	278.3728274	$RG_{ISI_{\left(p,q\right)}}\left(C_{6}\right)=6{\left[2\right]}^{q}$
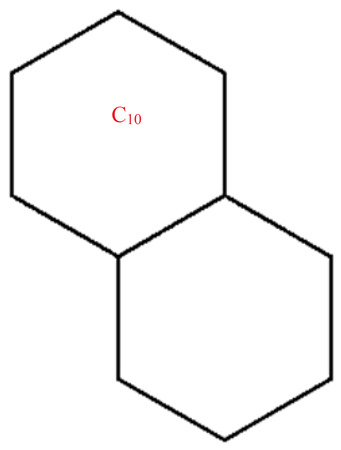	582.3543	$RG_{ISI_{\left(p,q\right)}}\left(C_{10}\right)={\left[2\right]}^{q}+{\left[2\right]}^{p+2}{\left[3\right]}^{q}+6{\left[4\right]}^{p+q}$
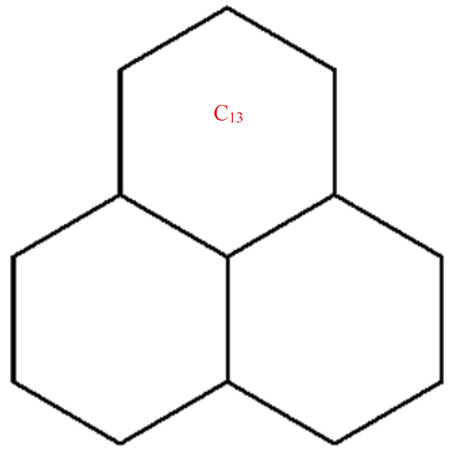	870.9878	$RG_{ISI_{\left(p,q\right)}}\left(C_{13}\right)=3{\left[2\right]}^{q}+{\left[2\right]}^{p+1}{\left[3\right]}^{q+1}+6{\left[4\right]}^{p+q}$
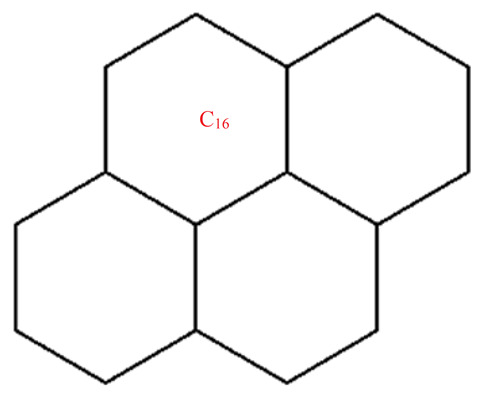	1165.387066	$RG_{ISI_{\left(p,q\right)}}\left(C_{16}\right)=5{\left[2\right]}^{q}+{\left[2\right]}^{p+3}{\left[3\right]}^{q}+6{\left[4\right]}^{p+q}$
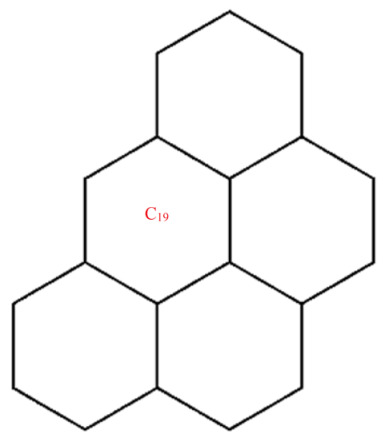	1512.946726	$RG_{ISI_{\left(p,q\right)}}\left(C_{19}\right)=7{\left[2\right]}^{q}+5{\left[2\right]}^{p+1}{\left[3\right]}^{q}+6{\left[4\right]}^{p+q}$
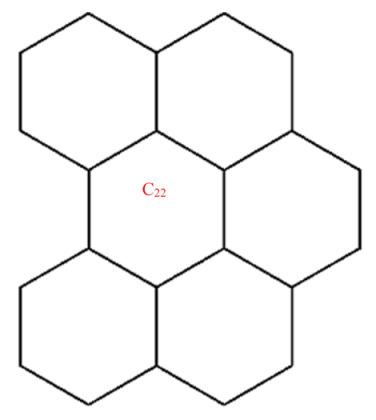	1868.115158	$RG_{ISI_{\left(p,q\right)}}\left(C_{22}\right)=5{\left[2\right]}^{q+1}+{\left[2\right]}^{p}{\left[3\right]}^{q+2}+2{\left[4\right]}^{p+q+1}$
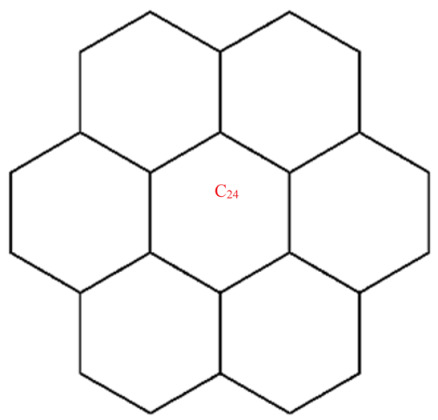	2129.987845	$RG_{ISI_{\left(p,q\right)}}\left(C_{24}\right)=3{\left[2\right]}^{q+2}+{\left[2\right]}^{p+2}{\left[3\right]}^{q+1}+6{\left[4\right]}^{p+q}$
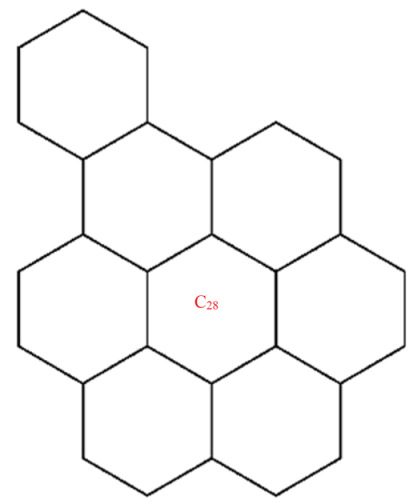	2698.081041	$RG_{ISI_{\left(p,q\right)}}\left(C_{28}\right)=15{\left[2\right]}^{q}+{\left[2\right]}^{p+2}{\left[3\right]}^{q+1}+2{\left[4\right]}^{p+q+1}$
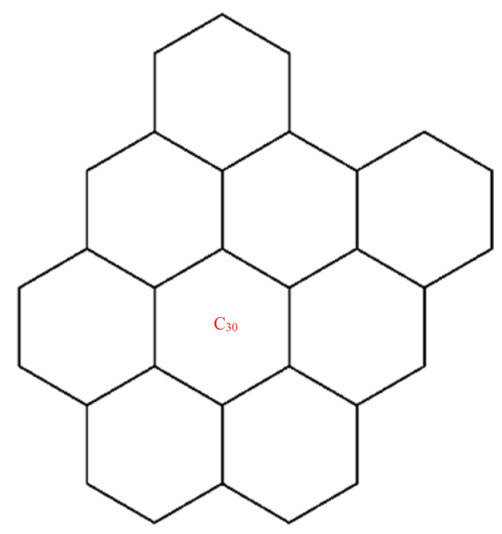	2951.482056	$RG_{ISI_{\left(p,q\right)}}\left(C_{30}\right)=17{\left[2\right]}^{q}+7{\left[2\right]}^{p+1}{\left[3\right]}^{q}+7{\left[4\right]}^{p+q}$
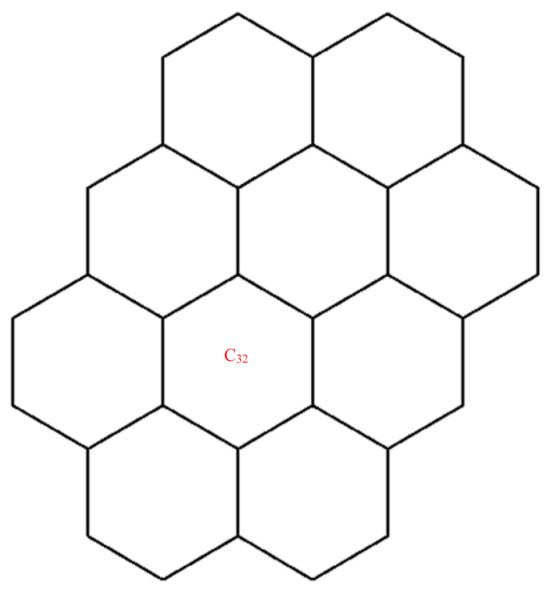	3253.425639	$RG_{ISI_{\left(p,q\right)}}\left(C_{32}\right)=19{\left[2\right]}^{q}+{\left[2\right]}^{p+4}{\left[3\right]}^{q}+6{\left[4\right]}^{p+q}$

**Table 4:** Linear prediction models with statistical parameters of the exchange-correlation energy of graphene sheets from *C*_6_ to *C*_32_.

Regression Equation	*r*	SE	*F*
$ECE=123.542+24.198(RM_{1}$)	0.980475724	213.8003228	198.893
$ECE=123.542+24.198(RM_{2}$)	0.945489731	354.0705116	67.437
$ECE=22.043+3.584(RR_{\frac{1}{2}}$)	0.997953668	69.52114251	1948.719
ECE=4.057+3.371(RSCI)	0.998617364	57.15508183	2887.026
ECE=4.057+3.371(RH)	0.997778424	72.43364998	1794.526
ECE=4.057+3.371(RHZ)	0.947685472	347.0617000	70.514
ECE=4.057+3.371(RGA)	0.997280715	80.12777418	1464.977
ECE=4.057+3.371(RAG)	0.980655201	95.36092088	1031.971
ECE=4.057+3.371(RISI)	0.980655201	212.8250165	200.793
$ECE=123.542+24.198(RReZG_{1}$)	0.998037144	68.08980614	2031.849
$ECE=123.542+24.198(RReZG_{3}$)	0.896497248	481.7127938	32.755

**Table 5:** Numerical values of the graphene for l>1,k>1.

(l, k)	$RM_{1}$	$RM_{2}$	RR	RSCI	RH	RHZ	RGA	RAG	RISI	$RReZG_{1}$	$RReZG_{3}$
(1, 1)	24	23	2.9142	2.9476	2.8333	94	5.8856	6.1213	5.8333	6	90
(2, 2)	58	45	13.657	11.154	13.333	188	18.542	19.485	13.833	28	154
(3, 3)	104	73	30.339	23.604	27.833	306	37.199	38.849	24.833	62	230
(4, 4)	162	107	53.142	40.295	52.333	448	61.856	64.213	38.833	108	318
(5, 5)	232	147	81.885	61.231	80.833	614	92.512	95.577	55.833	166	418
(6, 6)	314	193	116.63	86.408	115.33	804	129.17	132.94	75.833	236	530
(7, 7)	408	245	157.11	115.83	155.83	1018	171.83	176.30	98.833	318	654
(8, 8)	514	303	204.11	149.50	202.83	1256	220.48	225.67	124.83	412	790
(9, 9)	632	367	256.86	187.40	254.83	1518	275.14	281.03	153.83	518	938
(10, 10)	762	437	315.60	229.54	313.33	1804	335.80	342.40	185.83	636	1098

## Data Availability

All the raw data supporting the conclusion of this paper were provided by the authors.
